# Cautionary High-resolution Computed Tomography Findings for the Presence of Facial Canal Dehiscence in Patients with Cholesteatoma

**DOI:** 10.7759/cureus.6717

**Published:** 2020-01-21

**Authors:** Deniz Baklacı, İhsan Kuzucu, İsmail Guler, Rauf Oğuzhan Kum, Müge Özcan

**Affiliations:** 1 Otolaryngology, Kahramankazan State Hospital, Ankara, TUR; 2 Otolaryngology, Aksaray University Faculty of Medicine, Aksaray, TUR; 3 Otolaryngology, Medipol University School of Medicine, Ankara, TUR; 4 Otolaryngology, Ministry of Health Ankara City Hospital, Ankara, TUR

**Keywords:** facial canal dehiscence, cholesteatoma, chronic otitis media, computed tomography, predictive factor

## Abstract

Objective

This study aimed to investigate the abnormal high-resolution computed tomography (HRCT) findings in cholesteatomatous chronic otitis media (CCOM) patients preoperatively and the coexistence of abnormal HRCT findings with facial canal dehiscence (FCD) observed intraoperatively to identify the predictive factors associated with FCD.

Methods

The medical records of 151 CCOM patients who had undergone tympanomastoidectomy at our center were retrospectively examined in terms of the patients’ age and gender, preoperative HRCT findings [scutum defect, posterior wall of external auditory canal (PWEAC) defect, lateral semicircular canal (LSSC) defect, tegmen defect, and sigmoid plate erosion]. Operation records containing information about FCD were also analyzed.

Results

The prevalence of FCD was found to be 33.8% (51/151). There was a significant correlation between the presence of scutum, PWEAC, LSSC, and tegmen defects and the presence of FCD. However, no statistically significant correlation was found between the presence of sigmoid plate erosion and the presence of FCD. The results of regression analysis of the coexisting pathologic findings for FCD showed that the risk of FCD was highest in patients with LSSC + scutum defects (34.3-fold increase), followed by LSSC + PWEAC defects (31.6-fold increase).

Conclusion

Our study revealed that the presence of scutum, PWEAC, LSSC, and tegmen defects on HRCT indicates a higher risk of FCD preoperatively. This risk is even greater when multiple abnormal findings coexist.

## Introduction

Facial paralysis is one of the most feared complications encountered during cholesteatoma surgery. The rate of iatrogenic facial paralysis was reported as 15% in the mid-20th century [1]. With the development of high-magnification microscopes and motorized drills, this rate has been drastically reduced. Recently, the reported risk of facial injury is 0.6-3.6% for primary surgery and 4-10% for revision surgery [2].

The risk of injury during surgery is high in cases of dehiscence of the facial nerve (FN) canal [3,4]. Facial canal dehiscence (FCD) may be caused by developmental defects due to failure during the ossification process of the bony canal or bony erosion resulting from cholesteatoma and inflammation. The most common location is at the tympanic segment, with a majority of these dehiscences occurring through the inferior and underside of the FN adjacent to the oval window at the rates of 73.5% and 55%, respectively [5,6]. In the literature, the rate of FCD varies from 1 to 74% [5-9]. Li et al. have reported the rate of FCD as 11.4% in stapedectomies [10]. The incidence of FCD was also found to be high in patients with chronic otitis media (COM), ranging from 18 to 34% [3,11]. FCD was reported to occur at a higher rate during revision surgery than primary surgery [12]. Therefore, the status of the facial canal during COM surgery remains a challenging issue even for experienced surgeons. Intraoperatively, some pathological conditions such as scutum erosion, defects of the posterior wall of the external auditory canal (PWEAC), labyrinth fistula, complete ossicular chain defect, and the presence of cholesteatoma were found to be important signs for the prediction of FCD [13-15].

Iatrogenic FN damage may occur during the removal of cholesteatoma from the middle ear cavity, epitympanum, and mastoid cavity. Thus, it is essential to accurately evaluate the facial canal anatomy and its relationship with the surrounding pathology in these cases. Obtaining a clear picture of the ear anatomy based on detailed radiographic information concerning the bony canal of FN and determining the extension and site of cholesteatoma can minimize the likelihood of FN damage during surgery. Furthermore, FCD has been considered to be a negative prognostic factor for surgical outcome in COM surgery [16]. In this regard, high-resolution computed tomography (HRCT) of the temporal bone provides valuable data since it can determine the cholesteatoma tissue and its extent, and assess the ossicles, facial canal, tegmen, PWEAC, bony plate over the sigmoid sinus and jugular vein, carotid canal, and inner ear structures [17-19]. Low-lying dura or anteriorly located sigmoid sinuses, which are considered important in mastoid surgery, can also be visualized preoperatively via HRCT scanning. When the surgeon has good knowledge of temporal bone anatomy and its relationship with the adjacent structures mentioned above, s/he can decide on the type of surgery (i.e., open or closed technique) and inform the patient about possible complications. However, the sensitivity of HRCT is low in the detection of preoperative FCD; thus, some authors have suggested that routine preoperative HRCT examination prior to uncomplicated COM surgery was of questionable value [14,17,20,21]. Therefore, in addition to having good knowledge of anatomy, the surgeon must also consider the conditions in which the FCD rates may be high.

In the present study, we aimed to investigate the abnormal HRCT findings in cholesteatomatous COM (CCOM) patients preoperatively and the coexistence of abnormal HRCT findings with FCD observed intraoperatively. We strove to identify the predictive factors associated with FCD based on preoperative HRCT findings and facilitate preemptive prediction of FCD.

## Materials and methods

The medical records of 151 CCOM patients who had undergone tympanomastoidectomy at our center between 2014 and 2019 were reviewed. The data collected retrospectively included the patients’ age and gender, preoperative HRCT findings (scutum defect, PWEAC defect, lateral semicircular canal (LSSC) defect, tegmen defect, and sigmoid plate erosion. We also obtained operation records containing information about FCD. The exclusion criteria were having a history of COM surgery or temporal bone trauma, congenital cholesteatoma, middle ear pathologies except for COM, lack of preoperative HRCT scans, and presence of congenital inner ear anomalies.

Facial canal observations were performed by experienced surgeons using an operating microscope and confirmed by palpation with a blunt pick. Intraoperative FN monitoring was used only in complicated cases. The patients were categorized into two groups based on intraoperative observation of the facial canal: Group 1 with FCD and Group 2 without FCD. The groups were compared in terms of preoperative abnormal HRCT findings mentioned above.

The local ethics committee approved the research (2019/06-21). The study was performed in compliance with the Declaration of Helsinki. Written informed consent was obtained from all participants prior to surgery.

HRCT was performed using scanners with 64-detector rows (Toshiba Aquilion TSX-101A; Canon Medical Systems Corporation, Otawara, Japan) using the following scan parameters - detector collimation: 0.5 mm; tube voltage: 120 kV; tube current: 250 mA; and rotation time: 1.0 seconds.

Statistical analysis

Statistical Package for the Social Sciences (SPSS) software v. 20 (IBM Corporation, Armonk, NY) was used for statistical analyses. The data were expressed as mean and standard deviation (SD). The chi-square test was employed for the comparison of the groups. The Shapiro-Wilk test was used to check the normality of the data. The Student’s t-test was utilized for parametric variables. A logistic regression analysis was undertaken to investigate the predictive role of preoperative HRCT findings associated with FCD in univariate analyses. P-values of less than 0.05 were considered statistically significant.

## Results

A total of 151 patients with 151 operated ears (80 left, 71 right) who met the aforementioned criteria were evaluated. The demographic and clinical features of the patients are given in Table 1. There were no statistically significant differences between the two groups in terms of age (p:0.393) and gender (p: 0.670).

**Table 1:** Demographic and clinical characteristics of the study group FCD: facial canal dehiscence; PWEAC: posterior wall of external auditory canal; LSSC: lateral semicircular canal; SD: standard deviation

	Number (total: 151)	Percentage
Age, years (mean ±SD)	42.08 ±15.41	
Gender		
Female	66	43.7
Male	85	56.3
Side operated		
Left	80	52.9
Right	71	47.1
FCD		
Absent	100	66.2
Present	51	33.8
Scutum defect		
Absent	40	26.5
Present	111	73.5
PWEAC defect		
Absent.	45	29.8
Present	106	70.2
LSSC defect		
Absent	126	83.4
Present	25	16.6
Tegmen defect		
Absent	136	90.1
Present	15	9.9
Sigmoid plate erosion		
Absent	134	88.7
Present	17	11.3

There were 111 patients with scutum defects, and 48 of these patients (43.2%) had FCD. FCD without a scutum defect was determined in three of 40 patients (7.5%). The correlation between scutum defect and FCD was statistically significant [p: <0.001; odds ratio (OR): 9.39; 95% confidence interval (CI): 2.73-32.31] (Table 2). 

**Table 2:** Comparison of the groups in terms of abnormal HRCT findings HRCT: high-resolution computed tomography; FCD: facial canal dehiscence; PWEAC: posterior wall of external auditory canal; LSSC: lateral semicircular canal, SD: standard deviation; OR: odds ratio; CI: confidence interval

Clinical characteristics	FCD	WITHOUT FCD	OR	95% CI	P-value
Scutum defect, n (%)					
Absent	3 (7.5)	37 (92.5)	9.4	2.73–32.31	<0.001
Present	48 (43.2)	63 (56.8)			
PWEAC defect, n (%)					
Absent	8 (17.8)	37 (82.2)	3.1	1.34–7.44	0.009
Present	43 (40.6)	63 (59.4)			
LSSC defect, n (%)					
Absent	30 (23.8)	96 (76.2)	16.8	5.34–52.80	<0.001
Present	21 (84)	4 (16)			
Tegmen defect, n (%)					
Absent	42 (30.9)	94 (69.1)	3.3	1.12–10.04	0.024
Present	9 (60)	6 (40)			
Sigmoid plate erosion, n (%)					
Absent	42 (30.9)	92 (69.1)	2.4	0.89–6.83	0.076
Present	9 (52.9)	8 (47.1)			

A total of 106 patients had PWEAC defect, of whom 43 (40.6.%) had FCD. FCD without PWEAC defect was determined in eight of 45 patients (17.8%). The correlation between PWEAC defect and FCD was statistically significant (p: 0.007; OR: 3.16; 95% CI: 1.34-7.44).

LSSC defects were detected in 25 patients, of whom 21 (84.0%) had FCD. Thirty of 126 patients (23.8%) were found to have FCD without an LSSC defect. A statistically significant correlation was detected between LSSC defect and FCD (p: <0.001; OR: 16.80; 95% CI: 5.34-52.80).

There were 15 patients with tegmen defects, with nine of them (60.0%) having FCD. FCD without a tegmen defect was observed in 42 of 136 patients (30.9%). The correlation between tegmen defect and FCD was statistically significant (p: 0.024; OR: 3.36; 95% CI: 1.12-10.04).

Of the 17 patients with sigmoid plate erosion, nine (52.9%) had FCD. Forty-two of 134 patients (31.3%) had FCD without sigmoid plate erosion. There was no statistically significant correlation between sigmoid plate erosion and FCD (p: 0.076; OR: 2.46; 95% CI: 0.89-6.83). The results of the regression analysis for FCD in patients with coexisting HRCT abnormalities are given in Table 3.

**Table 3:** Results of regression analysis for FCD in patients with coexisting HRCT abnormalities HRCT: high-resolution computed tomography; FCD: facial canal dehiscence; PWEAC: posterior wall of external auditory canal; LSSC: lateral semicircular canal; OR: odds ratio; CI: confidence interval

	Number (%)	OR	95% CI	P-value
Scutum + LSSC	23 (15.2)	34.3	7.60–154.79	<0.001
PWEAC + LSSC	22 (14.6)	31.6	6.99–142.90	<0.001

## Discussion

Today, HRCT has become the most important and standard imaging modality in COM patients due to the detailed information it provides prior to surgical interventions on bony structures, including possible variations and complications. HRCT shows the localization and spread of pathological soft tissues in COM patients. It also allows visualization of the pathology of areas that cannot be examined otoscopically, such as the posterior tympanic area and facial recess.

Previous studies have demonstrated a good correlation between HRCT scans and surgical findings, particularly in scutum, ossicular chain and sigmoid plate erosion, and PWEAC and LSSC defects. However, there are concerns that HRCT lacks guaranteed sensitivity and specificity for the determination of FCD. In the literature, the sensitivity rates of HRCT for the FCD range from 0 to 100% while the reported specificity rates for FCD are considerably higher [19-27]. Tatlipinar et al. determined that the thin bone covering the FN was occasionally difficult to evaluate owing to partial volume averaging with adjacent soft tissue; thus, the authors were able to detect 35 of 39 (89.7%) FCD using HRCT with a negative predictive value of 89.7% [17]. Similarly, HRCT was reported to be unsuccessful in the determination of FCD by some authors. For example, O’Reilly et al. identified only four out of nine FCD cases, and Freng et al. one in four cases [28,29]. In contrast, O’Donoghue et al. successfully detected all nine of the FCD cases they investigated; however, they had six false positives [30].

**Figure 1:**
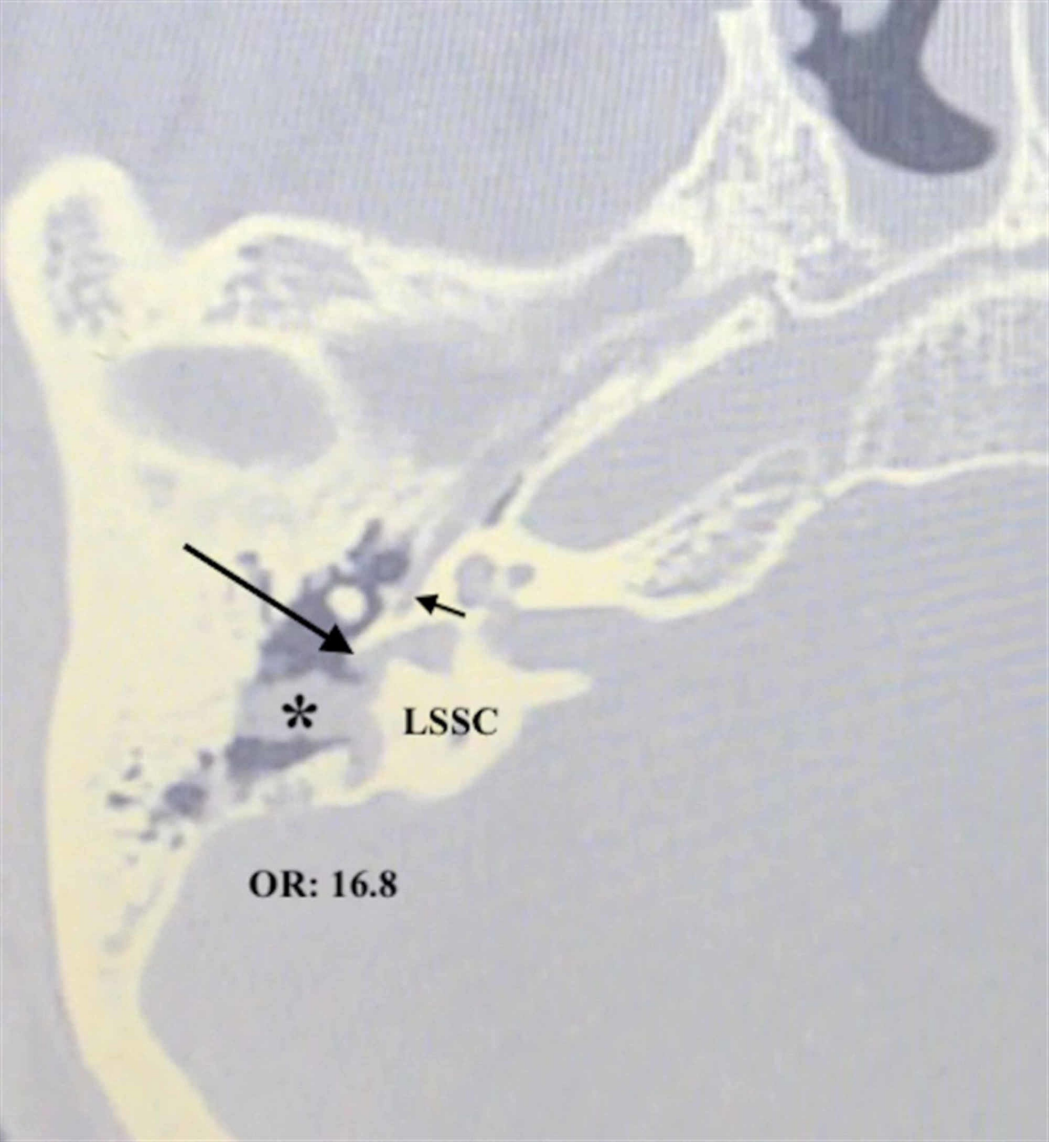
LSSC defect caused by cholesteatoma LSSC: lateral semicircular canal; OR: odds ratio *cholesteatoma Long arrow: labyrinth fistula caused by cholesteatoma; short arrow: facial canal dehiscence

Due to the relative low sensitivity of HRCT in detecting FCD compared to other anatomical temporal bone structures, some authors suggest using intraoperative findings to predict FCD with higher accuracy. In this regard, Ozbek et al. found that the presence of an LSSC defect or tegmen erosion of >1 cm significantly increases the risk of FCD. The risks for FCD were 22.5 and 12.06 times higher in patients with LSSC and tegmen defects, respectively [13]. Similarly, Gulustan et al. reported that the likelihood of FCD was significantly higher in patients with an LSSC defect (24.2-fold increase), PWEAC defect (4.1-fold increase), or a stapes defect [15]. In an HRCT study investigating the radiological and surgical correlation in patients with FCD and coexisting abnormalities, the authors reported that LSSC defect was highly associated with FCD [19]. Our results are consistent with previous studies since we also calculated the risk of FCD to be 16.8, 3.1 and 3.3 times higher in patients with LSSC (Figure 1), PWEAC, and tegmen defects, respectively. Additionally, when LSSC and PWEAC defects coexisted in a patient, the risk of FCD was 31.6 times higher.

**Figure 2:**
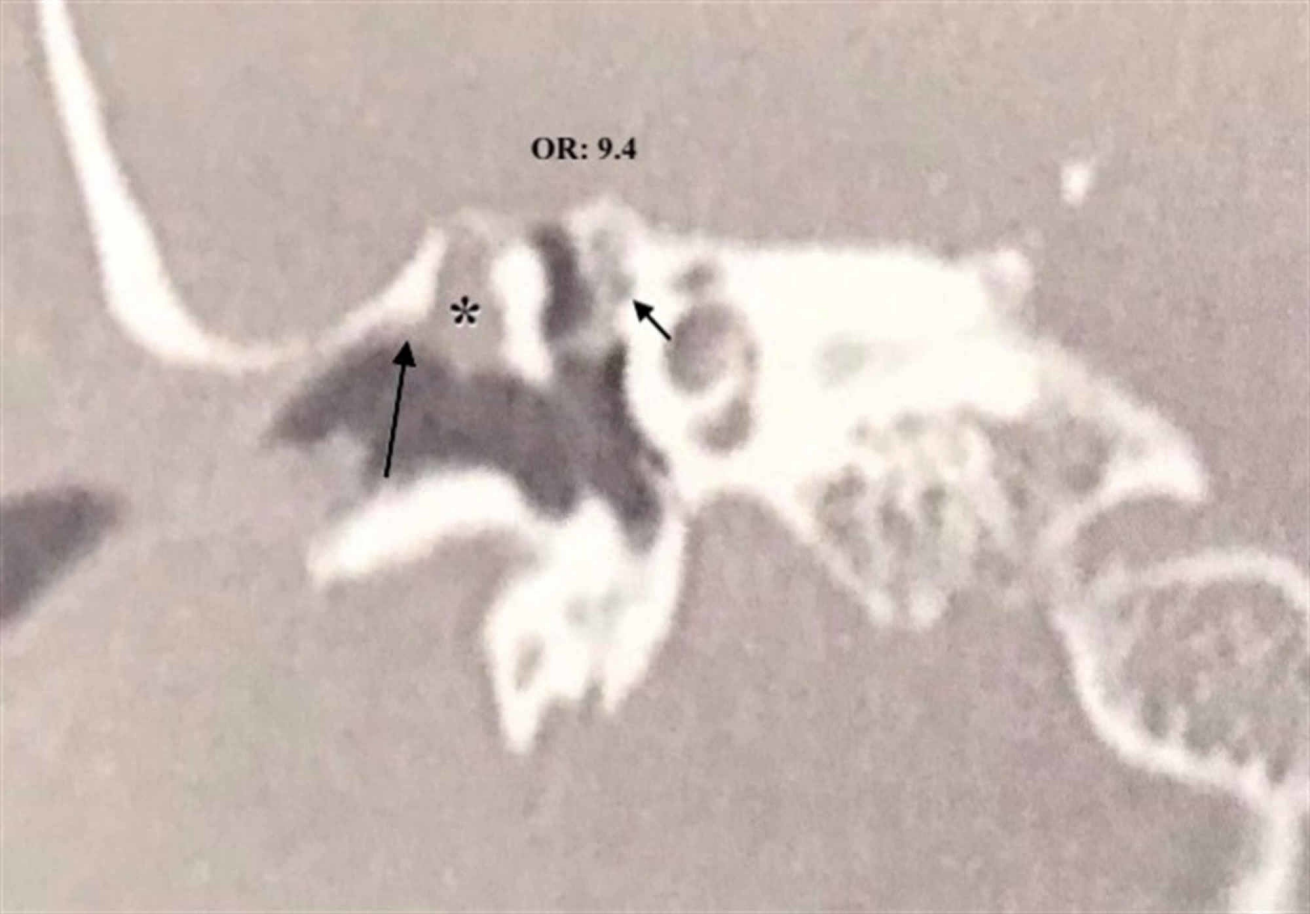
Scutum defect caused by cholesteatoma OR: odds ratio *cholesteatoma Long arrow: scutum defect; short arrow: facial canal dehiscence

Genc et al. observed FCD in 55.5% of patients with scutum defects [14]. In the present study, 43.2% of patients with scutum defects had FCD, indicating a 9.4 times higher risk compared to patients without a scutum defect (Figure 2). Therefore, preoperative indications about LSSC, PWEAC, and scutum defects should be considered as a warning sign for the presence of FCD.

In some CCOM patients, especially those with an extended disease, abnormal HRCT findings may coexist. In this study, the results of regression analysis of the coexisting pathologic findings for FCD showed that the risk of FCD was highest in patients with LSSC + scutum defects (34.3- fold increase), and LSSC + PWEAC defects (31.6- fold increase). It can be stated that as the cholesteatoma extends in the middle ear, the likelihood of FCD increases.

There are two limitations to be addressed in this study. Firstly, some personal biases may have crept in during the evaluation of HRCT scans or surgical findings. Secondly, in retrospective studies, some of the records obtained from medical charts may be incomplete or lost in the course of time, leading to missing data. Further prospective studies can provide more reliable and accurate data that could provide stronger evidence for the role and importance of HRCT in determining FCD.

## Conclusions

Our study revealed that the presence of scutum, PWEAC, LSSC, and tegmen defects on HRCT indicates a higher risk of FCD preoperatively. This risk is even greater when multiple abnormal findings coexist. Given that the imaging of the facial canal is much more difficult than imaging these structures, we believe that our findings are important for otologic surgery.
